# Supplementary figures and images for: The effect of mangrove restoration on avian assemblages of a coastal lagoon in southern Mexico
Source: PeerJ. 2019 Aug 13;7:e7493. doi: 10.7717/peerj.7493 (PMC6697041; doi:10.7717/peerj.7493)

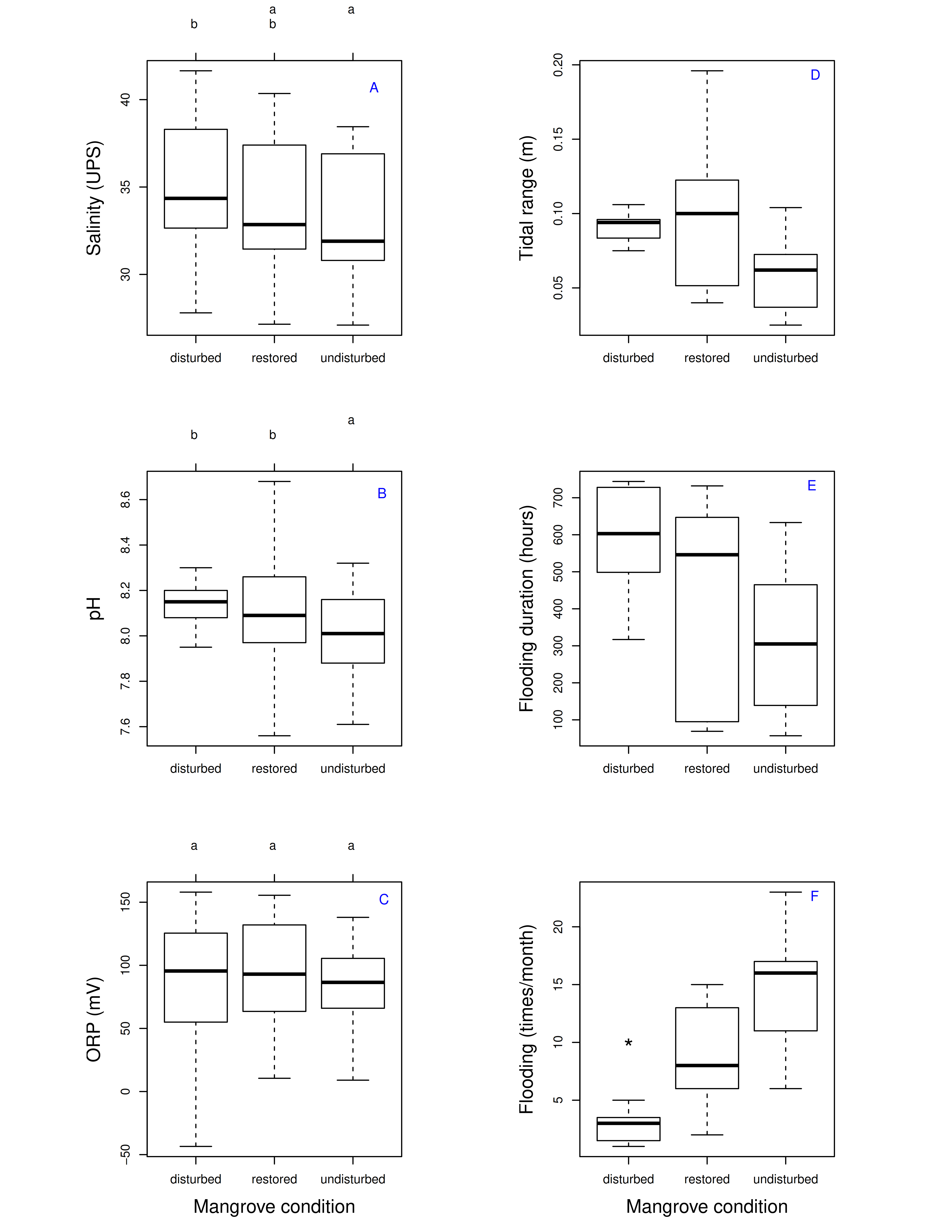

Supplement: Figure S1 — Same letters indicate no differences between the study areas. On the right side the hydroperiod components (D–F). The star represents significant differences among different mangrove conditions. The boxes show the inter-quartile range, the thick black lines within the boxes are the medians, and the whiskers are the minimum and maximum values. [file peerj-07-7493-s001.png]

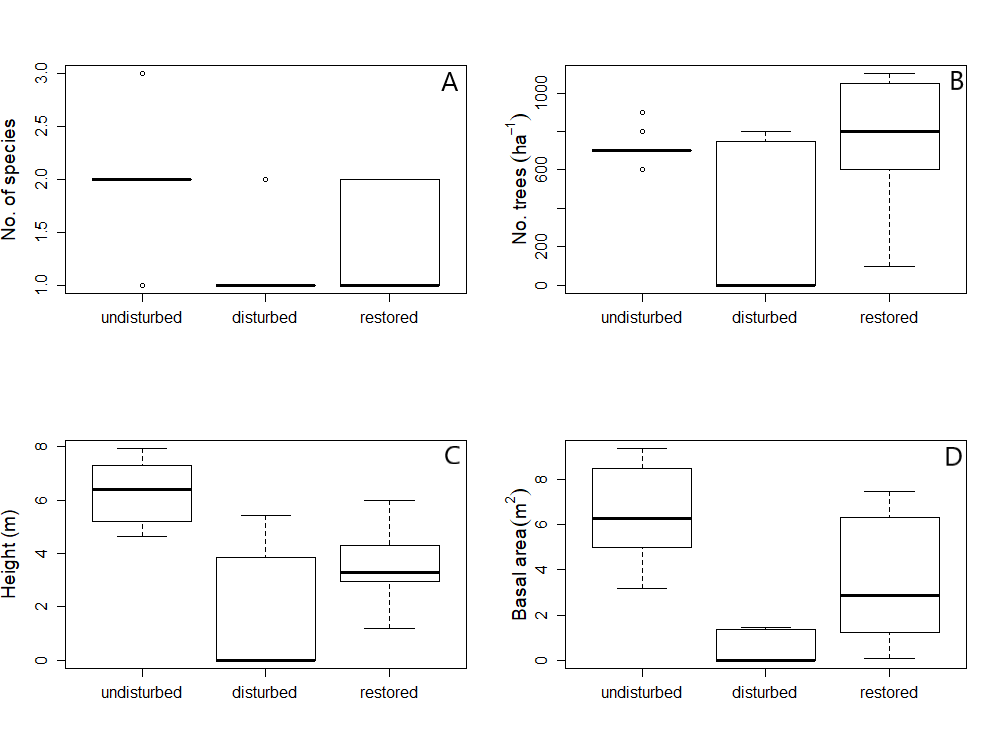

Supplement: Figure S2 — (A) show the number of tree species. In (B) the density of mangrove tress is shown. (C) shows the measured height of the mangrove trees at each sampling site. (D) shows the cover expressed as basal area at each sampling site. [file peerj-07-7493-s002.png]

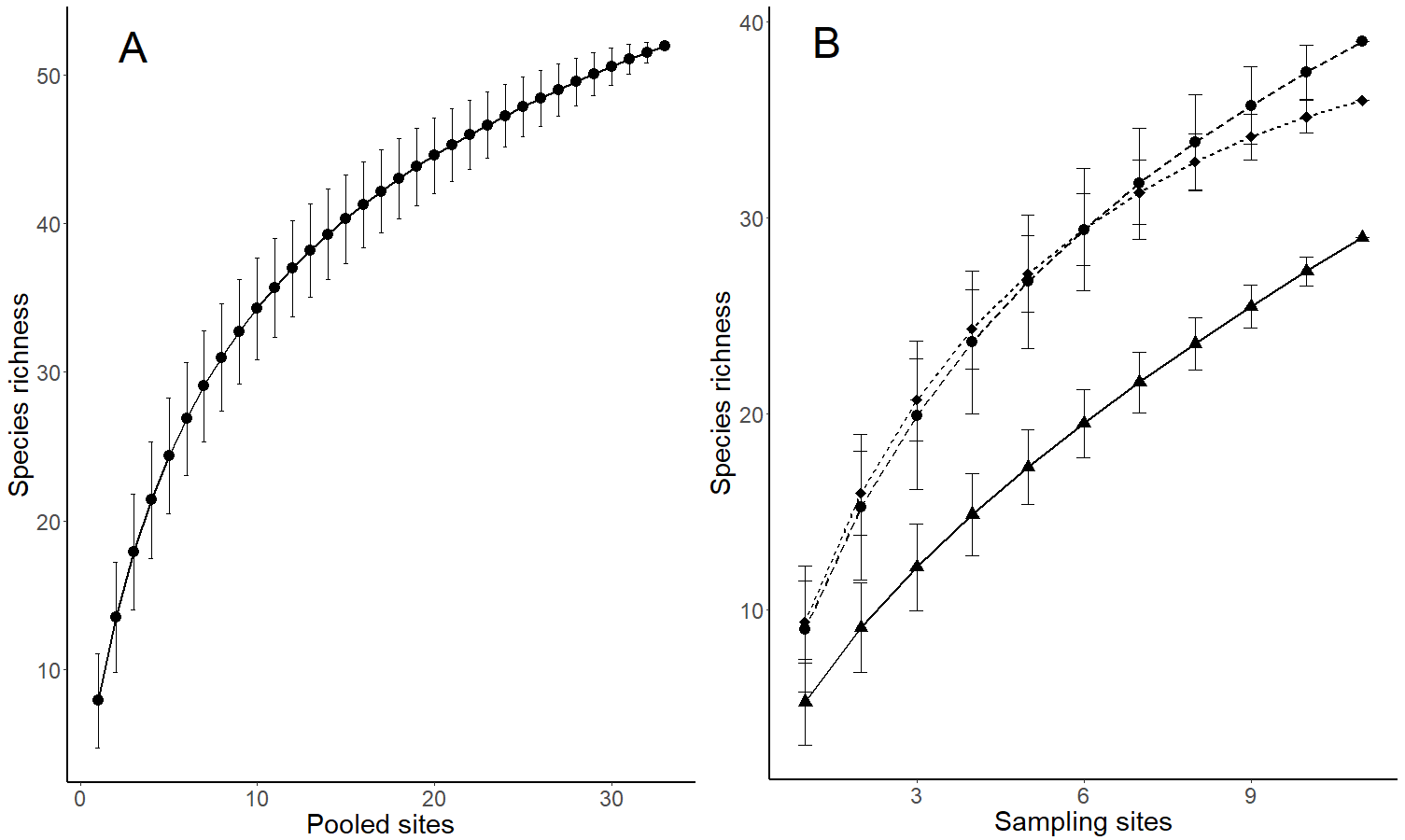

Supplement: Figure S3 — The different studied sites are disturbed (triangles), restored (crosses), and undisturbed (circles). In both graphics, bars indicate ±1.5 standard deviations. [file peerj-07-7493-s003.png]

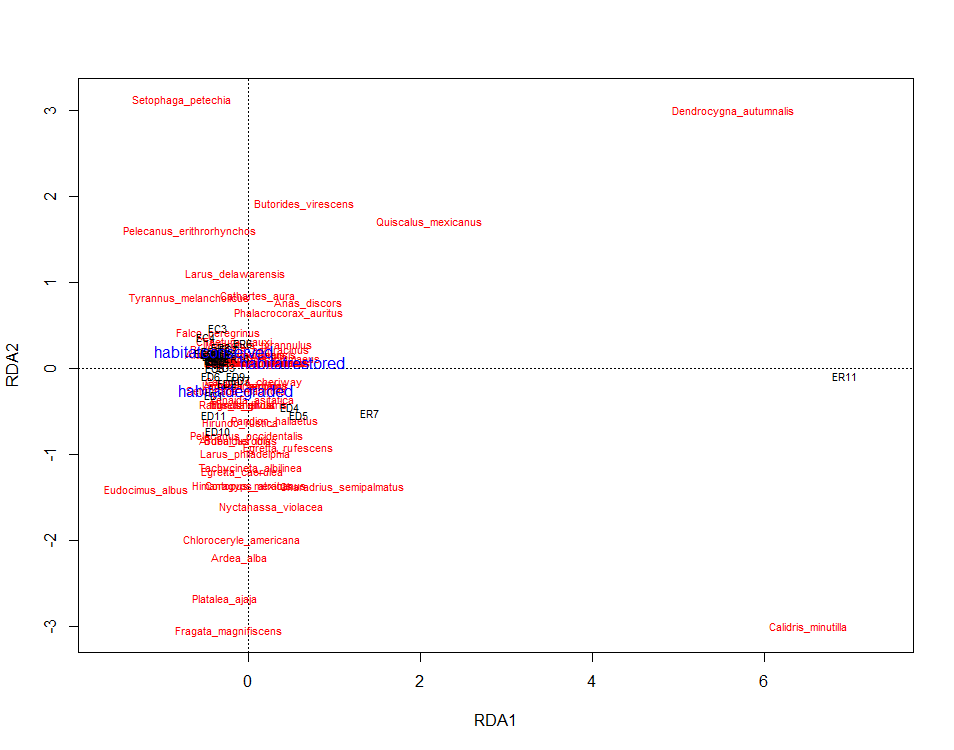

Supplement: Figure S4 [file peerj-07-7493-s004.png]
