# Supplementary material for: The effect of mangrove restoration on avian assemblages of a coastal lagoon in southern Mexico
Source: PeerJ. 2019 Aug 13;7:e7493. doi: 10.7717/peerj.7493 (PMC6697041; doi:10.7717/peerj.7493)
Supplement: Table S1 — Area: restored (R), undisturbed (U), disturbed (D) NOM-059: Is the list of threatened species recognized by the Mexican Environmetal Authorities. The categories are: least concern (LC), subject to special protection (SP), threatened (TH). Pop.Trend are the population trends as reported by the International Union for the Conservation of Nature (IUCN). [file peerj-07-7493-s005.pdf]

ORDER	Scientific name	Common name	Area			Migration	Conservation status		
			R	U	D		NOM-059	IUCN	Pop. Trend
**Anseriformes**	*Dendrocygna autumnalis*	Black-bellied Whistling-Duck				Resident	LC	LC	Increasing
	Anas discors	Blue-winged Teal				Winter visitor	LC	LC	Decreasing
**Ciconiiformes**	*Ardea alba*	Great Egret				Resident	LC	LC	Unknown
	Butorides virescens	Green Heron				Resident	LC	LC	Decreasing
	Mycteria americana	Wood stork				Winter visitor	SP	LC	Decreasing
	Eudocimus albus	White Ibis				Resident	LC	LC	Stable
	Nyctanassa violacea	Yellow-crowned Night-Heron				Resident	TH	LC	Stable
	Egretta caerulea	Little Blue Heron				Winter visitor	LC	LC	Decreasing
	Egretta thula	Snowny Egret				Resident	LC	LC	Increasing
	Platalea ajaja	Roseate Spoonbill				Resident	LC	LC	Stable
	Ardea herodias	Great Blue Heron				Winter visitor	LC	LC	Increasing
	Egretta rufescens	Reddish Egret				Resident	SP	NT	Increasing
	Egretta tricolor	Tricolored Heron				Winter visitor	LC	LC	Increasing
**Gruiformes**	*Rallus longirostris*	Mangrove Rail				Resident	SP	LC	Decreasing
**Suliformes**	*Fregata magnificens*	Magnificent Frigatebird				Winter visitor	LC	LC	Increasing
	Anhinga anhinga	Anhinga				Resident	LC	LC	Decreasing
	Phalacrocorax auritus	Double-crested Cormorant				Winter visitor	LC	LC	Increasing
**Pelecaniformes**	*Pelecanus erythrorhynchos*	American White Pelican				Winter visitor	LC	LC	Increasing
	Pelecanus occidentalis	Brown Pelican				Resident	LC	LC	Increasing
**Accipitriformes**	*Coragyps atratus*	Black Vulture				Resident	LC	LC	Increasing
	Buteogallus anthracinus	Common Black-Hawk				Resident	SP	LC	Increasing
	Pandion haliaetus	Osprey				Winter visitor	LC	LC	Increasing
	Cathartes aura	Turkey Vulture				Resident	LC	LC	Stable
**Falconiformes**	*Caracara cheriway*	Crested Caracara				Resident	LC	LC	Increasing
	Falco peregrinus	Peregrine Falcon				Winter visitor	SP	LC	Stable
**Charadriiformes**	*Calidris minutilla*	Least Sandpiper				Winter visitor	LC	LC	Decreasing
	Charadrius semipalmatus	Semipalmated Plover				Winter visitor	LC	LC	Stable
	Himantopus mexicanus	Black-necked Stilt				Resident	LC	LC	Increasing
	Larus argentatus	Herring Gull				Winter visitor	LC	LC	Decreasing
	Larus atricilla	Laughing Gull				Winter visitor	LC	LC	Increasing
	Haematopus palliatus	American Oystercatcher				Winter visitor	LC	LC	Stable
	Larus delawarensis	Ring-billed Gull				Resident	LC	LC	Increasing
**Culumbiformes**	*Patagioenas fasciata*	Band-tailed Pigeon				Resident*	LC	LC	Decreasing
	Zenaida asiatica	White-winged Dove				Winter visitor	LC	LC	Increasing
**Cuculiformes**	*Coccyzus minor*	Mangrove Cuckoo				Winter visitor	LC	LC	Stable
	Crotophaga sulcirostris	Groove-billed Ani				Resident	LC	LC	Decreasing
**Apodiformes**	*Chaetura vauxi*	Vaux's Swift				Resident	LC	LC	Increasing
	Amazilia rutila	Cinnamon Hummingbird				Resident	LC	LC	Unknown
**Coraciiformes**	*Chloroceryle aenea*	American Pygmy-kingfisher				Resident	LC	LC	Decreasing
	Chloroceryle americana	Green Kingfisher				Resident	LC	LC	Increasing
**Piciformes**	*Melanerpes pygmaeus*	Yucatan Woodpecker				Resident	LC	LC	Stable
	Setophaga petechia bryanti	Mangrove Warbler				Resident	LC	LC	Stable
	Tachycineta albilinea	Mangrove Swallow				Resident	LC	LC	Decreasing

**Passeriformes**	*Hirundo rustica*	Barn Swallow				Resident	LC	LC	Decreasing
	Mimus gilvus	Tropical Mockingbird				Resident	LC	LC	Increasing
	Quiscalus mexicanus	Great-tailed Grackle				Winter visitor	LC	LC	Stable
	Setophaga magnolia	Magnolia Warbler				Resident	LC	LC	Increasing
	Tyrannus melancholicus	Tropical Kingbird				Resident	LC	LC	Increasing
	Icterus cucullatus	Hooded Oriole				Resident	LC	LC	Increasing
	Myiarchus tyrannulus	Brown-crested Flycatcher				Winter visitor	LC	LC	Increasing
	Pitangus sulphuratus	Great Kiskadee				Resident	LC	LC	Increasing
	Setophaga citrina	Hooded Warbler				Resident	LC	LC	Increasing
	Empidonax virescens	Acadian flycatcher				Transient	LC	LC	Stable

Area: restored (R), undisturbed (U), disturbed (D)
NOM-059: Is the list of threatened species recognized by the Mexican Environmetal Authorities.
The categories are: least concern (LC), subject to special protection (SP), threatened (TH).
Pop.Trend are the population trends as reported by the International Union for the Conservation of Nature (IUCN)
