# Supplementary material for: The effect of mangrove restoration on avian assemblages of a coastal lagoon in southern Mexico
Source: PeerJ. 2019 Aug 13;7:e7493. doi: 10.7717/peerj.7493 (PMC6697041; doi:10.7717/peerj.7493)
Supplement: Table S2 — H, habitat condition; R, measured Redox; T, water temperature; S, water salinity; Sps, number of tree species; B, Basal area; Dn, trees density; Dt, detectability. [file peerj-07-7493-s006.docx]

| A) Model | T-statisc | p-value | AIC |
| --- | --- | --- | --- |
| H + B * Dn + R + T + S + Sps + B | 18.24 | 0.003 | 2321.908 |
| H * T + R + S + Sps + B + Dn | 18.83 | 0.001 | 2333.707 |
| H * R + T + S + Sps + B + Dn | 18.43 | 0.002 | 2355.120 |
| H * S + R + T + Sps + B + Dn | 18.33 | 0.002 | 2375.955 |
| H * B + R + T + S + Sps + Dn | 17.99 | 0.002 | 2397.705 |
| H * Dn + R + T + S + Sps + B | 17.86 | 0.003 | 2397.810 |
| H * Sps + R + T + S + B + Dn | 19.44 | 0.001 | 2400.829 |

| B) Model | T-statisc | p-value | AIC |
| --- | --- | --- | --- |
| Dt | 3.979 | 0.007 | 744.371 |
| H + Dt | 6.359 | 0.002 | 754.330 |
| H + Dt + B + Dn + Sps | 7.292 | 0.178 | 774.935 |
